# Tree Species Richness Promotes Invertebrate Herbivory on Congeneric Native and Exotic Tree Saplings in a Young Diversity Experiment

**DOI:** 10.1371/journal.pone.0168751

**Published:** 2016-12-16

**Authors:** Annika Wein, Jürgen Bauhus, Simon Bilodeau-Gauthier, Michael Scherer-Lorenzen, Charles Nock, Michael Staab

**Affiliations:** 1 Department of Nature Conservation and Landscape Ecology, Faculty of Environment and Natural Resources, University of Freiburg, Freiburg, Germany; 2 Department of Silviculture, Faculty of Environment and Natural Resources, University of Freiburg, Freiburg, Germany; 3 Centre for Forest Research, Université du Québec à Montréal, Centre-ville Station, QC H3C 3P8 Montréal, Canada; 4 Department of Geobotany, Faculty of Biology, University of Freiburg, Freiburg, Germany; USDA-ARS Fort Keogh Livestock and Range Research Laboratory, UNITED STATES

## Abstract

Tree diversity in forests is an important driver of ecological processes including herbivory. Empirical evidence suggests both negative and positive effects of tree diversity on herbivory, which can be, respectively, attributed to associational resistance or associational susceptibility. Tree diversity experiments allow testing for associational effects, but evidence regarding which pattern predominates is mixed. Furthermore, it is unknown if herbivory on tree species of native vs. exotic origin is influenced by changing tree diversity in a similar way, or if exotic tree species escape natural enemies, resulting in lower damage that is unrelated to tree diversity. To address these questions, we established a young tree diversity experiment in temperate southwestern Germany that uses high planting density (49 trees per plot; plot size 13 m^2^). The species pool consists of six congeneric species pairs of European and North American origin (12 species in total) planted in monocultures and mixtures (1, 2, 4, 6 species). We assessed leaf damage by leaf-chewing insects on more than 5,000 saplings of six broadleaved tree species. Plot-level tree species richness increased leaf damage, which more than doubled from monocultures to six-species mixtures, strongly supporting associational susceptibility. However, leaf damage among congeneric native and exotic species pairs was similar. There were marked differences in patterns of leaf damage across tree genera, and only the genera likely having a predominately generalist herbivore community showed associational susceptibility, irrespective of the geographical origin of a tree species. In conclusion, an increase in tree species richness in young temperate forests may result in associational susceptibility to feeding by generalist herbivores.

## Introduction

If and how biodiversity relates to ecological processes is a central question in ecology [1]. Driven by the emerging evidence that human habitat modification leads to species loss and alters ecosystem functionality [2], several large-scale biodiversity experiments have been established to investigate the relationship between plant diversity and ecological processes. Originally, those experiments focused on grassland communities (e.g. [3,4]) and revealed a general and positive effect of plant diversity on ecological processes [5]. Biodiversity experiments with trees have followed [6–8] to extend questions from grasslands to forests, which are globally important ecosystems covering 30% of the terrestrial land area and are critical for global physical processes [9]. Because of the structural complexity of forests, it is still unclear if conclusions on the relationship between plant diversity and ecosystem functioning based on grasslands hold for forests [10,11]. A topic often studied with biodiversity experiments is herbivory, which is a crucial ecological process in forests and other ecosystems [12,13]. While testing the effect of tree diversity on herbivory, we also tested for the effect of tree species origin, as both properties are directly related to human activities [2,14].

Despite considerable research effort, the sign of the relationship between tree diversity and herbivory remains controversial [15]. Empirical studies found negative (e.g. [16–18]), positive (e.g. [19–21]), or mixed (e.g. [22]) effects of tree diversity on herbivory. These discrepancies among studies can partly be explained by variation in associational effects among the tree species in a community [16,23–25], with the sign of the associational effect depending on the level of host specialization by the most-damaging herbivores. Associational resistance is indicated by a negative relationship between herbivory and tree diversity, because in species-rich tree communities, specialist herbivores will have a lower availability of host species than in pure stands [18,26]. In contrast, associational susceptibility is indicated by a positive herbivory-diversity relationship where the highest level of herbivory occurs in more diverse tree communities [20,27], because generalist herbivores will perform better and cause more damage when many different host plants are available. By consisting of preassigned, synthetic communities, tree diversity experiments provide opportunities to quantify associational effects and to test for relationships between tree species richness and herbivory (e.g. [21,22,28]).

Many tree species have been introduced outside their original ranges for use in forestry or as ornamental plants [14], but it is unknown if associational effects are similar for native and exotic tree species. One would expect that when simultaneously manipulating the diversity of native and exotic trees, the exotic species would have lower susceptibility to herbivores ([29,30], but see [31]), independent of tree diversity, while native species will show either associational resistance or associational susceptibility. Surprisingly, to the best of our knowledge, this has never been tested with congeneric pairs of native and exotic species in a controlled experimental setting.

We established a tree diversity experiment in southwestern Germany that specifically uses a species pool of congeneric European and North American tree species. To test if and how (associational resistance vs. associational susceptibility) tree species richness and geographic origin relate to leaf damage, we conducted an extensive survey of herbivory caused by leaf chewers, a dominant group of insect herbivores in temperate forests. We expected that leaf damage on the native tree species would be correlated with tree species richness, while the exotic tree species would have reduced leaf damage, and as opposed to the native tree species, leaf damage would be unrelated to tree species richness.

## Methods

### Study site

The study was conducted at the Freiburg field site of IDENT (International Diversity Experiment Network with Trees; [32]), which is part of TreeDivNet, the global network of tree biodiversity experiments [8]. Located in southwestern Germany (48°01'10"N / 7°49'37"E) at an elevation of about 240 m above sea level, the climate is oceanic (Cfb following Köppen climate classification) and warm for Central Europe, with 11.8°C mean annual temperature and 836 mm mean annual precipitation (period from 1990–2015). July and August are the warmest months (mean temperature 20.9°C each); January is the coldest month (mean temperature 3.0°C). All months are humid, with most precipitation falling in summer. The sandy-loamy soil of the field site is a partly anthropogenic disturbed and rather shallow (40 cm) Cambisol with high gravel content. The site was likely not continuously forested since the Middle Ages. More recent land use includes a period as a military area from 1888 until 1992 (including an airfield since World War I), followed by sheep grazing on the grassland that subsequently developed. The experiment is surrounded by extensively used grassland. Beyond the grassland to the North, South and West (c. 100 m) there are hedgerows and other woody vegetation, which is at the margins dominated by *Rubus* sp. and the exotic tree species *Robinia pseudoacacia* L. before a continuous deciduous forest begins. The afforested field site is not protected and privately owned by the University of Freiburg, who gave permission to conduct this work. The field studies did not involve endangered or protected species.

### Experimental design

The Freiburg IDENT is a replication of the experimental sites in Auclair (Québec, Canada) and Cloquet (Minnesota, USA) [32]. Central to the design of IDENT is the high density of tree individuals, which reduces the time required for interactions between trees and for the emergence of potential biodiversity effects on ecological processes compared to longer-term experiments with lower planting densities that are in line with standard forestry practices (see [10]). As in all tree diversity experiments, early studies are necessarily limited to saplings. However, it is the seedling and sapling stages that are decisive for the future life time of any tree individual (e.g. [33]), since if a tree dies as a sapling it will not be able to reproduce and will have zero fitness. Thus, recently planted tree diversity experiments are valuable as they allow investigating how biotic and abiotic processes influence those important life stages [32].

In November 2013, approximately 20,000 tree seedlings were planted in plots with seven rows and columns in a grid pattern at a distance of 45 cm (49 trees per plot; plot size 13 m^2^). Around each plot, a buffer zone of 90 cm was left, i.e. the outermost rows of two adjacent plots were 1.8 m apart. To minimize competition between weeds and tree saplings, the ground of every plot was covered with a layer (ca. 5 cm) of mulch (spruce and fir bark) in spring 2014. All plots were inspected twice during the growing seasons of 2014 and 2015 and plots with significant weed cover were weeded by hand. Weed necromass was always left in place. Supplemental mulching was added to a small number of plots with high weed cover in spring 2015, and in 10 plots with aggressive growth of rhizomatous grasses a water permeable weed barrier was added to the row spaces between trees.

The tree species pool consists of twelve species selected according to leaf habit and continent of origin. Six species originate from North America and six from Europe, with three gymnosperm and three angiosperm species from each continent (see [32]). Species belong to six genera, each with a North American (mentioned first) and a European representative: *Acer saccharum* Marshall, *A*. *platanoides* L., *Betula papyrifera* Marshall, *B*. *pendula* Roth, *Quercus rubra* L., *Q*. *robur* L., *Larix laricina* (Du Roi) K. Koch, *L*. *decidua* Mill., *Picea glauca* (Moench) Voss, *P*. *abies* (L.) H. Karst., *Pinus strobus* L., and *P*. *sylvestris* L. Natural history details for all tree species can be found in Table 1 of Tobner et al. [32]. Seeds of European species were from regional southwestern German provenances while North American species were from various localities across Germany. Seedlings were supplied by a local nursery, where they had been grown from seeds for a period from 1 to 3 years depending on species. Initial survival was assessed 8 months after planting in summer 2014. Overall mortality was 6.8% and not systematically related to the experimental treatments but differed among species. *Larix laricina* (22.9%), *Q*. *robur* (20.7%), and *P*. *sylvestris* (12.8%) had highest mortality, which was lower than 6.0% for the remaining species. Dead seedlings were replanted in fall 2014.

**Fig 1:**
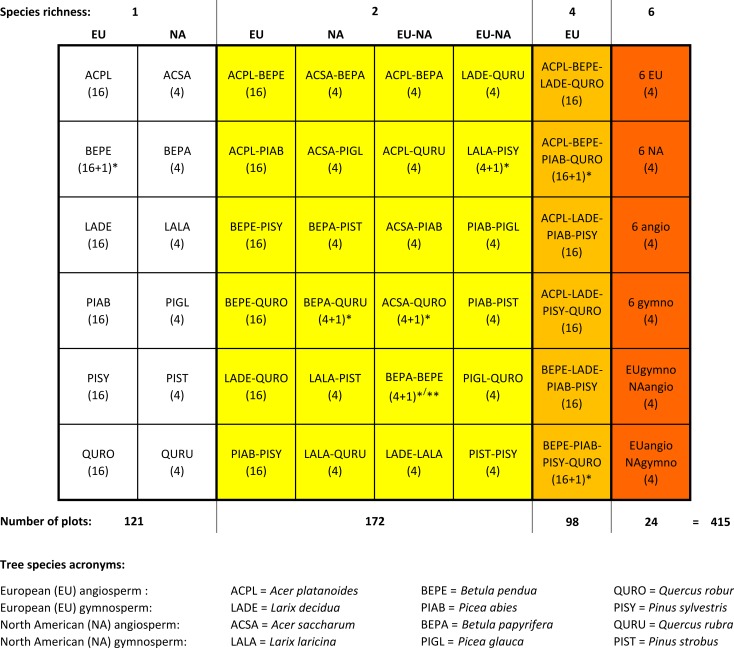
Schematic overview of tree species mixtures in Freiburg IDENT. Values in parentheses below species acronyms are the number of plots for each mixture. The experiment consists of 4 identical blocks with 102 plots each (S1 Fig). However, in block 4 seven mixtures (indicated by *) were duplicated, so in total there are 415 plots. Mixtures with 4 species were only established for European species. As a result, plots with purely European (EU) species are more frequent than plots with North American (NA) species or plots with species from both origins (EU-NA). Also, the fertilization treatment was only established for European species (see text). In block 3, the plot with both *Betula* species was mistakenly planted with both *Acer* species (indicated by **).

The experimental design comprises 4 replicated blocks with 102 plots each. In every block, there are 30 monoculture plots and 42, 24, and 6 plots with two, four, and six tree species each (see Fig 1 for detailed information on species compositions; see S1 Fig for a schematic map of Freiburg IDENT). Block 4 contains 7 additional plots (total number of plots in block 4: 109; total number of plots 415) outside the strict design; including data from those plots into all analyses does not affect the results. The presence of natives vs. exotics and angiosperms vs. gymnosperms was balanced as much as possible among the mixtures. Space limitations precluded the planting of four species mixtures containing North American tree species. Positions of plots in blocks were completely randomized but identical mixtures were not allowed to be direct neighbors. Tree positions in a plot were also randomized with a number of conditions to prevent strong differences in neighborhoods within plots among mixtures: 1) planting patterns (species adjacency) for each unique mixture varied with block; 2) tree numbers per species were equal in the outer two rows and the inner core of 5 x 5 trees; 3) when the number of trees per species per plot was uneven, the species with more individuals alternated between blocks; 4) no tree had four conspecific direct neighbors; 5) no grouping of 4 individuals (2 x 2 trees) of the same species within a plot; 6) in 4 and 6 species mixtures, a maximum of two contiguous individuals of the same species.

In the full design, mixtures comprising European species (with the exception of 6-species mixtures) were replicated for a tree species richness x fertilization sub-experiment with N, P, and N+P addition. In this study, we sampled all plots regardless of assignment to fertilization treatments because data collection was completed before the first fertilizer application of 2015 and, as expected, herbivory did not differ among treatments. The design of experiments in the IDENT network also aims at disentangling the effects of functional and species diversity among tree species mixtures (see Figure 1 in Tobner et al. [32]). While this rationale is fully implemented in Freiburg IDENT, e.g. by mixing angiosperms with gymnosperms (S1 Fig), we opted to use tree species richness to represent tree species diversity in lieu of functional diversity derived from leaf traits. We did so because leaf traits on tree saplings can have large intraspecific variation [34], which has not yet been quantified.

### Data collection

We expected that effects of tree species richness on herbivory would be stronger in the spring during and right after the leaf flushing period, since in temperate forests newly-emerged leaves are most attractive to herbivores because of their relatively lower tannin and higher nitrogen concentrations [35]. Therefore, sampling was done in spring, from 20-Apr-2015 to 21-May-2015. Data collection was limited to the six angiosperm species, as it is difficult to reliably assess herbivory on gymnosperm needles in a standardized way that is rapid enough to permit assessing the tree species richness gradient (see [36]). To minimize potential effects of adjacent mixtures on the target mixture, sampling was restricted to the central 5 x 5 tree individuals, resulting in a theoretical sample size of 5,430 trees. However, due to some mortality (mostly caused by root-feeding voles) and planting substitutions, 2.7% of the tree individuals could not be assessed, restricting sampling to 5,281 trees (Table 1). At the time of sampling, all species were established well and had grown to saplings. Leaf flushing of the three deciduous angiosperm genera is not fully synchronic in the study area: while *Betula* and *Acer* were flushing at the beginning of the sampling, *Quercus* flushed a few days later towards the end of April. Therefore, our sampling was temporally stratified, and plots containing *Quercus* individuals were only sampled once all *Quercus* individuals had flushed.

**Table 1:** Mean leaf damage by chewing insects per tree species richness level for the six studied tree species.

Tree species richness	*A*. *platanoides*	*A*. *saccharum*	*B*. *papyrifera*	*B*. *pendula*	*Q*. *robur*	*Q*. *rubra*	Total
1	0.48 ± 1.46 (393)	0.51 ± 1.73 (100)	0.97 ± 1.29 (100)	1.20 ± 1.11 (424)	1.24 ± 3.58 (372)	2.28 ± 4.96 (98)	1.03 ± 2.50 (1487)
2	0.50 ± 1.40 (503)	0.86 ± 2.15 (218)	1.30 ± 1.55 (258)	1.69 ± 1.73 (638)	1.39 ± 4.01 (482)	1.62 ± 2.88 (212)	1.24 ± 2.50 (2311)
4	1.62 ± 2.29 (391)	not planted	not planted	2.53 ± 2.33 (396)	0.94 ± 2.30 (399)	not planted	1.70 ± 2.40 (1186)
6	2.15 ± 2.46 (49)	1.49 ± 1.78 (49)	3.64 ± 2.36 (48)	3.20 ± 2.52 (48)	0.77 ± 1.82 (49)	2.04 ± 3.94 (54)	2.20 ± 2.76 (297)
Total	0.89 ± 1.85 (1336)	0.85 ± 2.01 (367)	1.50 ± 1.79 (406)	1.82 ± 1.88 (1506)	1.19 ± 3.37 (1302)	1.86 ± 3.71 (364)	1.34 ± 2.51 (5281)

Shown are absolute means per tree induvial (± standard deviation). Values in parentheses state the number of surveyed tree individuals. *Acer platanoides*, *Betula pendula*, and *Quercus robur* are native to Europe. *Acer saccharum*, *Betula papyrifera*, and *Quercus rubra* are native to North America.

Total damage caused by leaf-chewing herbivores, a dominant group of herbivorous insects in temperate forests, was visually estimated on ten leaves per tree individual (over 50,000 leaves in total). We used six a priori defined percentage classes (0%, < 5%, < 25%, < 50%, < 75%, > 75%, with mean values per class used in data analyses), a common approach for the standardized and rapid assessment of leaf damage (e.g. [21]). To rule out potential observer-specific sampling bias, the herbivory assessments were completed by one observer (A. Wein). The accuracy of the visual damage classes was satisfactorily high, as confirmed by scanning ten leaves of each damage class for each tree species and exactly measuring the missing leaf area using ImageJ (www.imagej.net) (see [19]).

### Data analyses

All analyses were conducted at the level of individual trees, and mean leaf-chewing herbivory was calculated from the ten assessed leaves per tree. To improve the normality and homoscedasticity of the data, mean herbivory values were log(x+1)-transformed and all statistical analyses were only performed with the transformed data.

Linear mixed-effects models fitted by maximum likelihood (R-package ‘lme4’, [37]) were used to test for the effect of tree species richness and geographic origin of a given tree species (Europe / North America) on leaf damage. Besides tree species richness and geographic origin, herbivory might depend on the total number of broadleaf trees per plot, assuming that herbivores feeding on angiosperms rarely feed on gymnosperms [38,39]. Also, in seasonal ecosystems, leaf damage on deciduous tree species will accumulate with ongoing season [17]. To account for these possible effects, we respectively included the fixed effects proportion of gymnosperms per plot and time of sampling (as Julian day) in all models. The interaction between tree species richness and Julian day was included to account for possible biases arising by the slightly delayed sampling of plots with *Quercus* (see above).

The IDENT experiment has a nested design with single trees being nested in plots and single plots being nested in blocks. Potential pseudoreplication arising from the experimental design [40] was addressed by appropriate consideration of the data structure, i.e. treating plot identity nested in block identity as a random effect in all models to account for possible block and plot specific variation in herbivory [41]. Similarly, herbivory per tree individual (the analyzed data unit) might be influenced by the specific neighborhood in a plot and strongly vary among different tree species (e.g. [21,42]). Thus, mixture identity and tree species identity were added as additional, non-nested random effects. To test if the results were influenced by gymnosperm species, we reanalyzed the entire dataset by replacing tree species richness (which includes gymnosperms) with the species richness of angiosperms. To test whether herbivores differentially damaged individual native and exotic species, we calculated species-specific models with species richness as the only fixed effect. Differences in leaf damage among congeneric species pairs and among genera were tested with models having tree species (or genus) identity as the only fixed effect and by subsequent post-hoc multiple comparisons of group-specific means (‘glht’ command in R-package 'multcomp', [43]). With the obvious exception of tree species identity, all those more specific models had the same random effect structure as above. Including this random effect structure across all models helped to account for systematic variation in the data potentially caused by the experimental design.

To find minimal, most-parsimonious models with the lowest number of fixed effects, we used a global model selection approach for all models containing more than one fixed effect (‘dredge’ command, R-package ‘MuMIn’, [44]). In brief, this procedure computes all possible candidate models based on the fixed effects of the full model and ranks best performing minimal candidate models by lowest AICc (corrected Akaike Information Criterion). In case two candidate models were equally likely (ΔAICc < 2), the more parsimonious model with the lower number of fixed effects was chosen. Degrees of freedom for calculating *P*-values of linear mixed-effect models were approximated after Kenward and Roger [45] (R-package ‘pbkrtest’, [46]). *R*^*2*^ values for all minimal models were computed with the methods suggested by Nakagawa and Schielzeth [47] as conditional *R*^*2*^ (variance explained by the fixed and random effects) and marginal *R*^*2*^ (variance explained by the fixed effects). Residuals of all linear mixed-effects models were checked for assumptions of normality and heteroscedasticity.

## Results

Damage by leaf-chewing insects affected 25.5% of the 52,810 leaves (raw data are in S1 File). Per tree sapling, chewing insects had consumed 1.3 ± 2.5% (mean ± SD) of leaf tissue, with up to 51.0% mean leaf damage per individual. The most-parsimonious model for leaf damage retained the fixed effects ‘tree species richness’ and ‘Julian day’ (Table 2; full models are presented in S1 Table) and explained 22.7% of the total variance (marginal *R*^*2*^). In comparison, about the same amount of variance was explained by the random effects (conditional *R*^*2*^ = 44.3%).

**Table 2:** Results of the best-performing linear mixed-effect for leaf damage by chewers.

Variable	Estimate ± SE	*t*	*P*
Julian day	0.041 ± 0.002	25.433	< 0.001
Tree species richness	0.023 ± 0.010	2.267	0.026

Shown are model estimates (i.e. slopes; SE = standard error), *t*-values and associated *P*-values. Analyses are based on log(x+1)-transformed data, and a global model selection approach was used to simplify the model.

**Fig 2:**
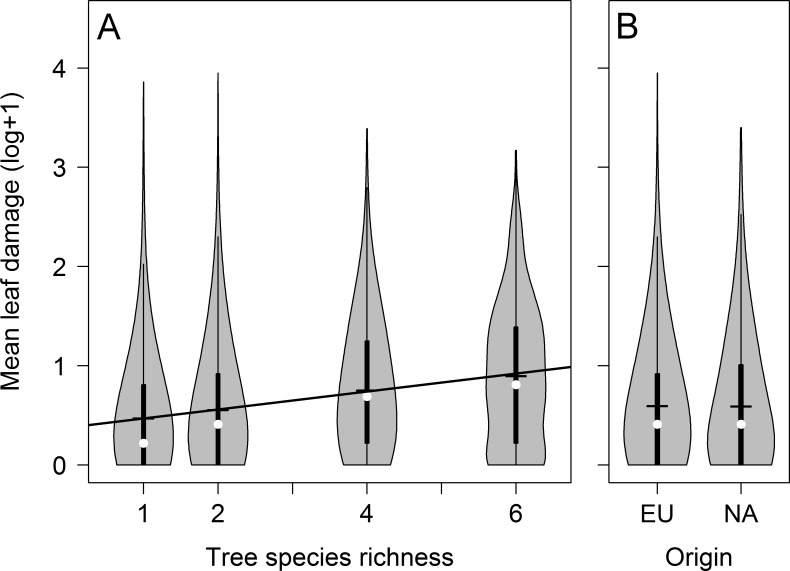
Leaf damage by chewing insects. (A) Relationship between leaf damage and tree species richness. The solid line illustrates the prediction of a linear model (significant at *P* < 0.05). (B) Comparison of leaf damage on native European (EU) and exotic North American (NA) trees. The data are log(x+1)-transformed mean damage values per tree individual and presented as violin plots that combine a boxplot with a kernel density plot; medians and means are, respectively, indicated by white dots and horizontal black lines.

Tree species richness had a significant and positive effect on herbivory by leaf chewers (*P* = 0.026) (Fig 2A; see Table 2 for detailed statistical output). Mean leaf damage in plots with six species (2.2 ± 2.8%) was more than twice as large as in monocultures (1.0 ± 2.5%) (Table 1). Contrary to our expectations, native European and exotic North American tree species had basically identical mean leaf damage (1.3 ± 2.5% vs. 1.4 ± 2.7%; Fig 2B) and origin was not included in the most-parsimonious model.

Herbivory increased significantly with the advancing spring during the data collection period (*P* < 0.001) (see S2 Fig). While we found < 1.0% removed tissue per tree in late April, the damage increased to > 2% by mid- May. The interaction between tree species richness and Julian day was not retained in the minimal, most parsimonious models, indicating that the positive effect of tree species richness on leaf damage was independent of sampling date. Patterns of leaf damage were invariant to the presence of gymnosperm trees in a plot and reanalysis of the data using angiosperm species richness instead of total species richness (see S2 Table and S3 Fig) provided similar results to the tree species richness results reported in the main text.

**Fig 3:**
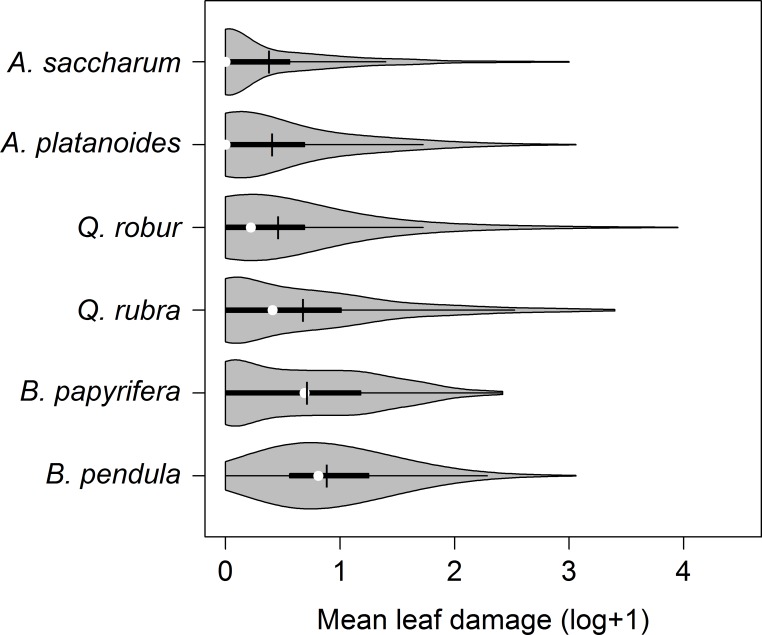
Leaf damage per tree species. All six investigated deciduous tree species are ordered from top to bottom by increasing mean values of log(x+1)-transformed leaf damage. See Fig 2 for further explanations.

**Fig 4:**
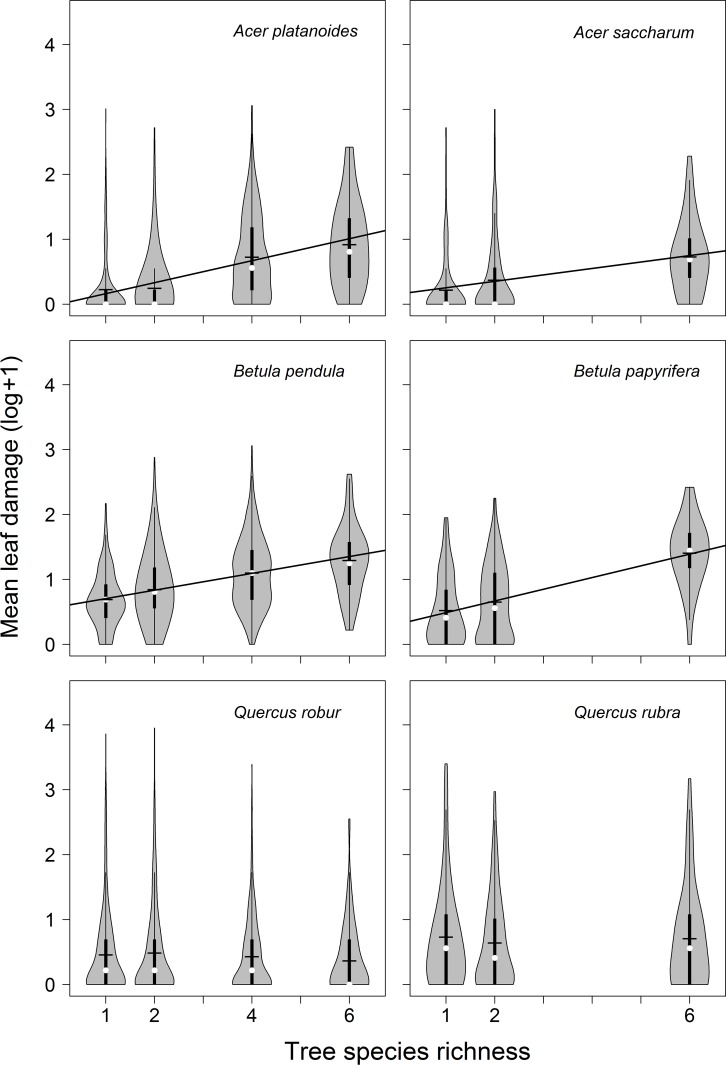
Relationships between tree species richness and mean leaf damage per tree species. Left and right panels show native European and exotic North American tree species for each genus pair, respectively. Shown are log(x+1) transformed values of mean leaf damage. Regression lines indicate significant relationships at *P* < 0.05. See Fig 2 for further explanations.

Leaf damage differed between tree genera and species. Interestingly, when arranging the six species by log(x+1)-transformed mean damage they assort by genera (Fig 3). Native and exotic species pairs in the same genus had consistently similar leaf damage, but differences between genera were highly significant (*P* < 0.001 for each combination of genera). Damage was highest on *Betula*, intermediate on *Quercus*, and lowest on *Acer* (Fig 3). When analyzed separately, all tree species except the two *Quercus* showed a significant and positive correlation between herbivory and tree species richness (*A*. *platanoides*: *P* = 0.013; *A*. *saccharum*: *P* = 0.011; *B*. *papyrifera*: *P* = 0.001; *B*. *pendula*: *P* = 0.006) (see Table 3 for details; Fig 4).

**Table 3:** Results of the species-specific linear mixed-effect models.

Species	Estimate ± SE	*t*	*P*
*Acer platanoides*	0.146 ± 0.053	2.801	0.013
*Acer saccharum*	0.102 ± 0.032	3.182	0.011
*Betula papyrifera*	0.189 ± 0.043	4.422	0.001
*Betula pendula*	0.122 ± 0.037	3.255	0.006
*Quercus robur*	-0.017 ± 0.015	-1.166	0.291
*Quercus rubra*	0.005 ± 0.026	0.134	0.897

Shown are model estimates (i.e. slopes ± SE), *t*-values and associated *P*-values for univariate models analyzing the relationship between species-specific leaf damage (log(x+1)-transformed data) and tree species richness. See also Fig 4.

## Discussion

Using data from saplings of deciduous trees growing in a controlled tree diversity experiment, we found a positive influence of plot-level tree species richness on leaf damage (i.e. associational susceptibility) that was surprisingly independent of the geographic origin of the assessed species. Levels of leaf damage were already in a range that can substantially reduce tree growth if occurring chronically over several years [48], but lower than in most related studies (e.g. [18,20–22,49]), likely because the data collection was carried out relatively early in the season. Nevertheless, by studying herbivory on congeneric native and exotic tree species simultaneously, our results contribute to the lively debate on the direction of the tree diversity-herbivory relationship (e.g. [22,23,28]).

### Associational effects as drivers of herbivory

The influence of plant species richness on herbivory can theoretically be described by associational resistance [26] or by associational susceptibility [27]. These related but opposing theories are supported by a theoretical framework [25], and both have empirical support (resistance: e.g. [16,18]; susceptibility: e.g. [19,20]). However, the proximate factors that may drive the diverging results between studies have rarely been explored (but see [24]). Whether associational resistance or associational susceptibility prevail when tree species richness changes depends largely on tree density, tree identity, and herbivore ecology [23,25,50].

Monocultures or low diversity tree communities provide easy to locate but homogeneous food resources to herbivores. If the most damaging herbivores are specialized on the respective tree species, a high resource density in low compared to high diversity plots will result in associational resistance [51]. In the present study a reduction in herbivory was not observed; our results support the associational susceptibility theory, which predicts that the most damaging herbivores are generalist feeders not affected by shifts in tree composition (but see [20]). Under high local tree diversity, generalists can spill over and feed on multiple host plants, as long as tree species are phylogenetically similar [39]. The resulting dietary mixing can strongly enhance the performance of generalist herbivores [52,53], likely caused by improved nutrient uptake and the dilution of secondary metabolites that would reach harmful concentrations in a homogenous diet [54,55]. However, we do not know if associational susceptibility in our study system also occurred in herbivore guilds that we did not assess, such as root feeder or sap sucker, or if it is restricted to leaf chewers. Also, arthropod communities in synthetic tree communities established on previously treeless land (i.e. most tree diversity experiments) may predominately consist of generalist herbivores, which may favor the detection of associational susceptibility over associational resistance.

Changing tree species richness may also affect higher trophic levels. It has been postulated (‘enemies hypothesis’, [51]) and shown (e.g. [56,57]; but see [58]) that higher plant diversity increases the diversity and abundance of natural enemies feeding on herbivores. From a theoretical perspective such top-down effects could offset associational susceptibility by reducing herbivore loads. However, detailed data on the arthropod community (including if exotic herbivores occur) are not yet available for the Freiburg IDENT experiment, and future research will test whether tree species richness affects natural enemies and possibly top-down regulation of herbivores.

### Differences in herbivory and associational susceptibility among tree species and genera

In tree diversity experiments, the identity of the studied tree species might be as or even more important than plot-level tree species richness to explain leaf damage [8]. One mechanism underlying the relationship between plant diversity and ecological processes in biodiversity experiments is the selection effect (see. e.g. [59]), i.e. the higher probability to include a plant species that dominates the studied ecological process, in high diversity mixtures. In herbivory studies, a selection effect may arise when there are species in the species pool that are especially prone to herbivore damage. A trees’ susceptibility to herbivores depends on many properties, such as secondary metabolites, local abundance, geographic origin or climatic niche marginality [42]. We found that leaf damage differs among tree species, which is commonly observed (e.g. [21,42]. *Betula papyrifera* and *B*. *pendula*, the species with the highest mean leaf damage in our study, exhibited positive correlations between damage and tree species richness. Finding a positive herbivory-richness correlation for the most-damaged tree species provides evidence that a selection effect was unlikely.

*Betula* species are typical early-successional taxa in temperate forests characterized by efficient resource utilization, rapid growth, and early reproduction [60]. Such tree species may invest less in defense against herbivores as they can compensate for the loss of leaf tissue by their quick compensatory growth [61]. In contrast to *Betula*, leaf damage was lowest in *A*. *platanoides* and *A*. *saccharum*. *Acer* leaves contain comparatively high amounts of phenolics involved in anti-herbivore defense, which might explain the low level of observed leaf damage [62]. *Quercus robur* and *Q*. *rubra* were the only two tree species on which leaf damage did not increase with tree species richness, conforming to other studies that found no [24] or negative [18] effects of tree species richness on leaf damage in *Quercus* saplings.

Interestingly, the observed tree species-specific presence or absence of associational susceptibility corresponds well to the feeding ecology of herbivorous insects found on the three genera in Central Europe. While temperate leaf-chewing insects are relatively generalistic [63], the herbivorous beetle fauna on *Quercus* consists of many species specialized on this genus. The number of specialist herbivores on *Acer* and *Betula* [64–66] is much lower. Recalling that the associational susceptibility theory builds on generalized herbivores and assuming that patterns of beetle specialization are similar for other herbivorous insect taxa (following Sprick and Floren [66]), this large-scale difference in herbivore ecology could possibly explain the non-consistent effect of tree species richness on leaf damage in IDENT as well as other tree diversity experiments. Likewise, associational susceptibility is unlikely for experiments comprising many tree species with a specialized herbivore community such as the ORPHEE experiment that has a species pool dominated by *Quercus* spp. (see [24]).

### Exotic tree species do not escape herbivores

For plant species introduced to novel areas, the ‘enemy release hypothesis’ [29] postulates lower herbivore damage in the novel compared to the native distribution ranges. Of the tree species studied by us, two have established outside their native ranges after they were initially transferred for use in forestry. The North American *Q*. *rubra* is the most abundant exotic hardwood species in Germany [67], has a long history of cultivation in forests, and is considered to have negative impacts on the forest vegetation in some European localities [68]. The European *A*. *platanoides* has successfully invaded woodlands in North America, and, in contrast to our results, Cincotta et al. [69] showed that the latter species has in North America lower leaf damage than its native congener *A*. *saccharum*. Furthermore, a recent assessment across continents provided evidence that escape and release from herbivores facilitated the invasion of *A*. *platanoides*, as leaf-damage was consistently lower in its novel compared to its native range [70].

By using a species pool of congeneric European and North American tree species pairs, Freiburg IDENT also transferred species to novel areas, but leaf damage within each genus was similar. Here exotic species did not escape herbivores (compare [31,71,72]). The concept of enemy release relies, among others, on two main criteria. First, herbivores causing the majority of damage are specialized. Second, exotic species are not too similar to the resident natives. Thus, enemy release is expected to be more pronounced for exotic plants lacking closely related native species [73], which was not the case in our experiment. Here exotics in novel ranges are frequently attacked by generalists or by herbivores spilling over from native plants having similar physical and chemical properties [38,71,72]. Furthermore, non-native trees are likely to be used as a food resource by generalist herbivores with a history of using related native trees as a food resource [74]. As explained above, the prevalent herbivores at our study site were probably generalists. Consequently, leaf-chewing insects in our experiment could cause similar damage to the exotic trees as to the neighboring native congeners [75]. This suggests that North American and European tree species had similar herbivores (albeit we lack data on herbivore identities), and explains our main result: associational susceptibility occurred and was independent of geographic origin found for both, native European and exotic North American tree species.

## Supporting Information

S1 Fig Schematic map of the Freiburg IDENT site. Different colors code for tree species richness. Plot number, fertilizer treatment and species mixtures per plot are indicated for each plot.
(PDF)

S2 Fig Relationship between Julian day and leaf damage by chewing insects. The regression line indicates a significant relationship at *P* < 0.05. The data are log(x+1)-transformed mean damage values per tree individual. See Fig 1 for further explanations.
(TIFF)

S3 Fig Relationship between angiosperm species richness and mean leaf damage. The regression line indicates a significant relationship at *P* < 0.05. The data are log(x+1)-transformed mean damage values per tree individual. See Fig 1 for further explanations.
(TIFF)

S1 File Raw data used for the analyses. (XLSX)

S1 Table Results of the full linear mixed-effect models. Shown are model estimates (± SE = standard error), *t*-values and associated *P*-values. Analyses are based on log(x+1)-transformed data.
(DOCX)

S2 Table Results of the linear mixed-effect models for angiosperm species richness instead of tree species richness. Shown are model estimates (± SE = standard error), *t*-values and associated *P*-values for the full model and the best-performing model after global model selection. Variables not included in the best-performing model are indicated with a dash. Analyses are based on log(x+1)-transformed data.
(DOCX)
