# Supplementary material for: Tree Species Richness Promotes Invertebrate Herbivory on Congeneric Native and Exotic Tree Saplings in a Young Diversity Experiment
Source: PLoS One. 2016 Dec 16;11(12):e0168751. doi: 10.1371/journal.pone.0168751 (PMC5161486; doi:10.1371/journal.pone.0168751)
Supplement: S1 Fig — Different colors code for tree species richness. Plot number, fertilizer treatment and species mixtures per plot are indicated for each plot. (PDF) [file pone.0168751.s001.pdf]

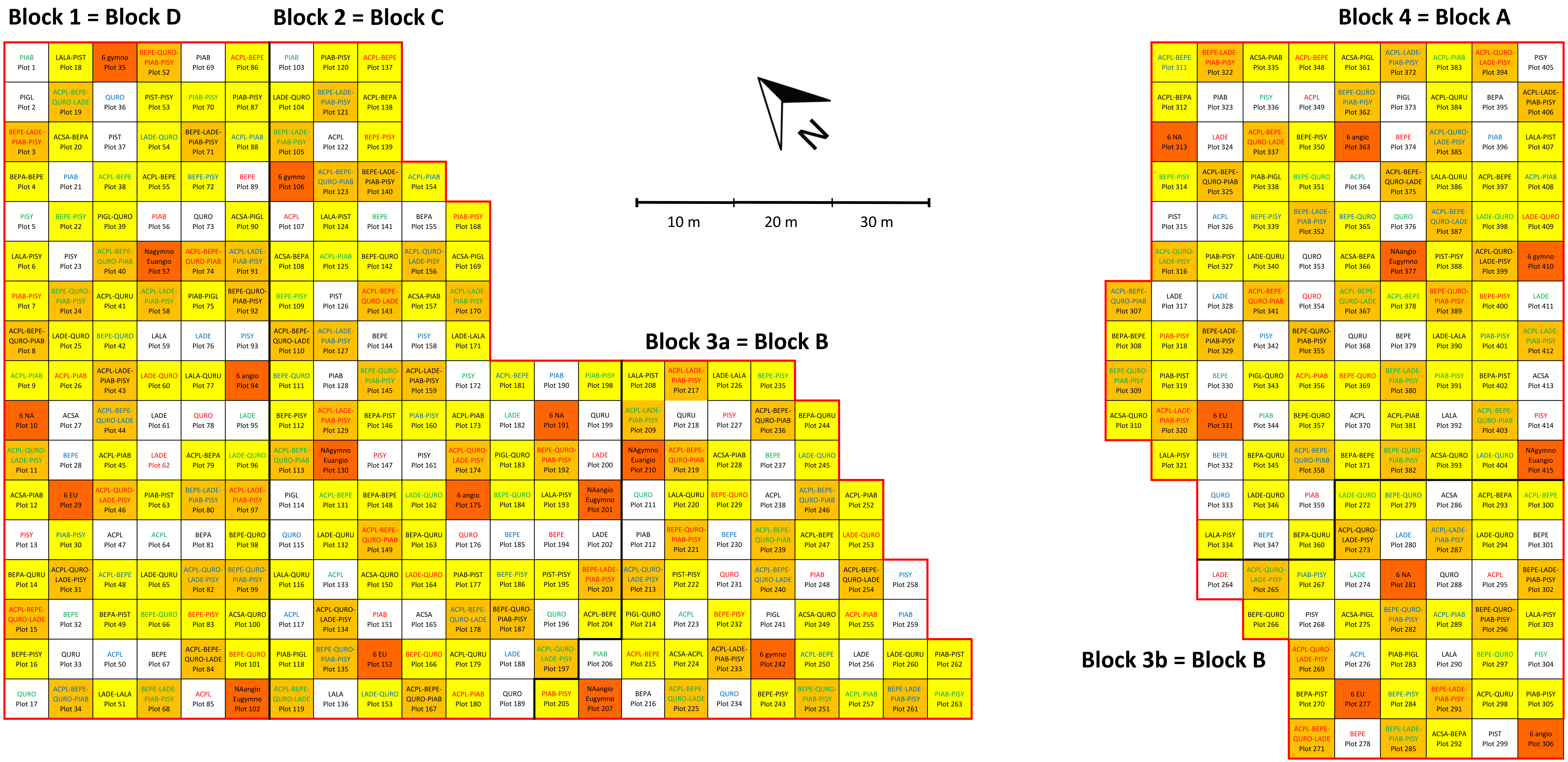

Fertilization

Black lettering = non fertilized plots
Blue lettering = N fertilized plots
Green lettering = P fertilized plots
Red lettering = N+P fertilized plots

Species richness

Monoculture
2-species mixture
4-species mixture
6-species mixture

Tree species

European (EU) angiosperm :	ACPL = *Acer platanoides*	BEPE = *Betula pendua*	QURO = *Quercus robur*
European (EU) gymnosperm:	LADE = *Larix decidua*	PIAB = *Picea abies*	PISY = *Pinus sylvestris*
North American (NA) angiosperm:	ACSA = *Acer saccharum*	BEPA = *Betula papyrifera*	QURU = *Quercus rubra*
North American (NA) gymnosperm:	LALA = *Larix laricina*	PIGL = *Picea glauca*	PIST = *Pinus strobus*

Figure S1 Schematic map illustrating the design of Freiburg IDENT. The experiment is located at 48°01'10"N / 7°49'37"E. Please see the legend for further explanations.
