# Supplementary figures and images for: Tree Species Richness Promotes Invertebrate Herbivory on Congeneric Native and Exotic Tree Saplings in a Young Diversity Experiment
Source: PLoS One. 2016 Dec 16;11(12):e0168751. doi: 10.1371/journal.pone.0168751 (PMC5161486; doi:10.1371/journal.pone.0168751)

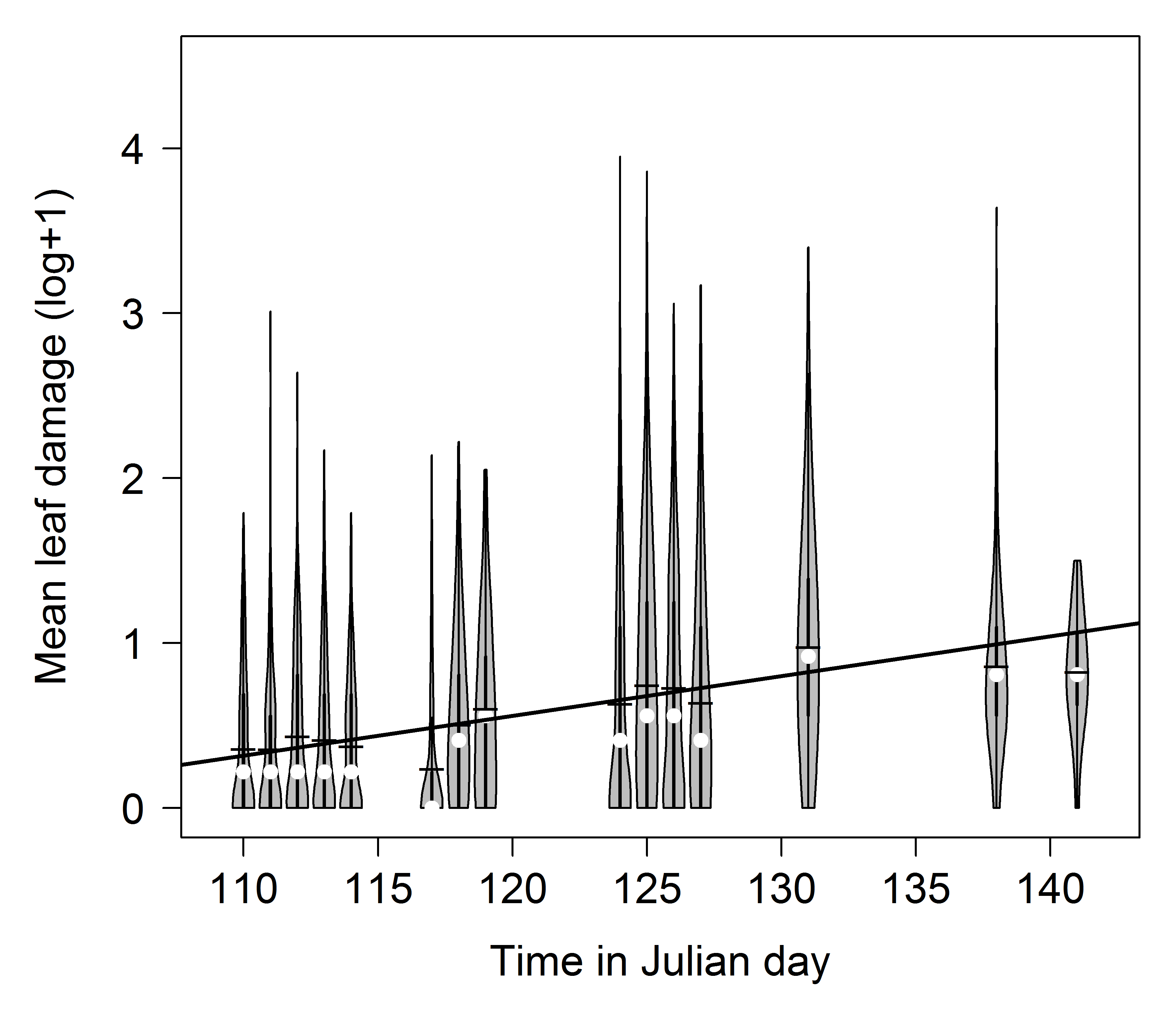

Supplement: S2 Fig — The regression line indicates a significant relationship at P < 0.05. The data are log(x+1)-transformed mean damage values per tree individual. See Fig 1 for further explanations. (TIFF) [file pone.0168751.s002.tiff]

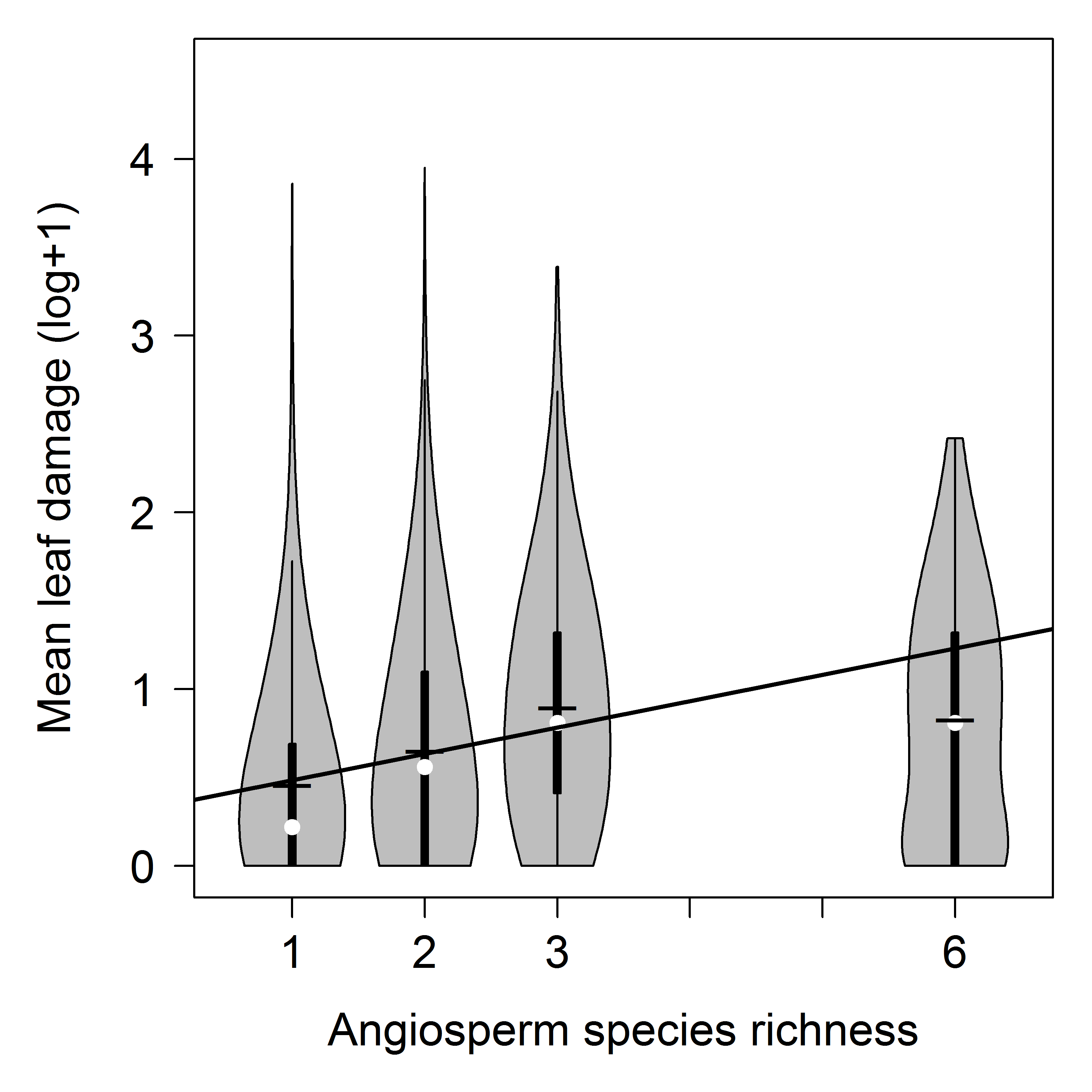

Supplement: S3 Fig — The regression line indicates a significant relationship at P < 0.05. The data are log(x+1)-transformed mean damage values per tree individual. See Fig 1 for further explanations. (TIFF) [file pone.0168751.s003.tiff]
