# Supplementary material for: Tree Species Richness Promotes Invertebrate Herbivory on Congeneric Native and Exotic Tree Saplings in a Young Diversity Experiment
Source: PLoS One. 2016 Dec 16;11(12):e0168751. doi: 10.1371/journal.pone.0168751 (PMC5161486; doi:10.1371/journal.pone.0168751)
Supplement: S1 Table — Shown are model estimates (± SE = standard error), t-values and associated P-values. Analyses are based on log(x+1)-transformed data. (DOCX) [file pone.0168751.s005.docx]

**Supporting Information for**

**Tree species richness promotes invertebrate herbivory on congeneric native and exotic tree saplings in a young diversity experiment**

Annika Wein, Jürgen Bauhus, Simon Bilodeau-Gauthier, Michael Scherer-Lorenzen, Charles Nock, and Michael Staab

**S1 Table. Results of the full linear mixed-effect models.**

| Variable | Estimate ± SE | *t* | *P* |
| --- | --- | --- | --- |
| Julian day | 0.041 ± 0.003 | 13.113 | < 0.001 |
| Tree species richness | 0.036 ± 0.152 | 0.233 | 0.816 |
| Origin North America | 0.005 ± 0.249 | 0.019 | 0.985 |
| Proportion gymnosperms | -0.106 ± 0.066 | -1.607 | 0.110 |
| Julian day:tree species richness | < 0.001 ± 0.001 | -0.023 | 0.982 |

Shown are model estimates (± SE = standard error), *t*-values and associated *P*-values. Analyses are based on log(x+1)-transformed data.
