# Supplementary material for: Tree Species Richness Promotes Invertebrate Herbivory on Congeneric Native and Exotic Tree Saplings in a Young Diversity Experiment
Source: PLoS One. 2016 Dec 16;11(12):e0168751. doi: 10.1371/journal.pone.0168751 (PMC5161486; doi:10.1371/journal.pone.0168751)
Supplement: S2 Table — Shown are model estimates (± SE = standard error), t-values and associated P-values for the full model and the best-performing model after global model selection. Variables not included in the best-performing model are indicated with a dash. Analyses are based on log(x+1)-transformed data. (DOCX) [file pone.0168751.s006.docx]

**Supporting Information for**

**Tree species richness promotes invertebrate herbivory on congeneric native and exotic tree saplings in a young diversity experiment**

Annika Wein, Jürgen Bauhus, Simon Bilodeau-Gauthier, Michael Scherer-Lorenzen, Charles Nock, and Michael Staab

**S2 Table. Results of the linear mixed-effect models for angiosperm species richness instead of tree species richness.**

| Variable | Estimate ± SE | *t* | *P* | Estimate ± SE | t | *P* |
| --- | --- | --- | --- | --- | --- | --- |
|  | Full model | | | Best-performing model | | |
| Julian day | 0.042 ± 0.003 | 12.206 | < 0.001 | 0.041 ± 0.002 | 25.572 | < 0.001 |
| Angiosperm species richness | 0.062 ± 0.247 | 0.251 | 0.802 | 0.036 ± 0.015 | 2.382 | 0.019 |
| Origin North America | 0.003 ± 0.250 | 0.010 | 0.992 | - | - | - |
| Proportion gymnosperms | 0.008 ± 0.059 | 0.143 | 0.887 | - | - | - |
| Julian day:angiosperm species richness | < 0.001 ± 0.002 | -0.103 | 0.918 | - | - | - |

Shown are model estimates (± SE = standard error), *t*-values and associated *P*-values for the full model and the best-performing model after global model selection. Variables not included in the best-performing model are indicated with a dash. Analyses are based on log(x+1)-transformed data.
